# Does chlorhexidine improve outcomes in non-surgical management of peri-implant mucositis or peri-implantitis?: a systematic review and meta-analysis

**DOI:** 10.4317/medoral.23633

**Published:** 2020-07-19

**Authors:** Siyan Liu, Min Li, Jianfeng Yu

**Affiliations:** 1 Department of Stomatology, affiliated Hospital of Shaoxing University, Shaoxing, Zhejiang, P.R. China

## Abstract

**Background:**

With greater number of implants being placed in clinical practice, incidence of peri-implant diseases are on the rise. It is not known whether chlorhexidine (CHX) improves outcomes in the management of peri-implant diseases. The aim of this systematic review and meta-analysis was to evaluate the role of CHX in improving outcomes with non-surgical management of peri-implant mucositis and peri-implantitis.

**Material and Methods:**

An electronic search of PubMed, Scopus, Embase, and CENTRAL (Cochrane Central Register of Controlled Trials) databases up to 1st August 2019 was carried out to search for studies evaluating the efficacy of CHX for non-surgical management of peri-implant diseases.

**Results:**

Seven studies were included. Four studies evaluated the role of CHX in peri-implant mucositis and three in peri-implantitis. Oral prophylaxis with mechanical cleansing of implant surface prior to CHX use was carried out in all seven studies. Meta-analysis indicated that use of CHX did not improve probing depths in peri-implant mucositis (SMD= 0.11; 95% CI: -0.16 to 0.38; *p*=0.42, I2= 0%). Similarly, CHX did not significantly reduce probing depths in patients with peri-implantitis (MD= 1.57; 95% CI: -0.88 to 4.0; *p*=0.21, I2= 98%). Results on the efficacy of CHX in reducing BOP in peri-implantitis are conflicting.

**Conclusions:**

Results of our study indicate that adjunctive therapy with CHX may not improve outcomes with non-surgical management of peri-implant mucositis. Conclusions with regards to its role in non-surgical management of peri-implantitis cannot be drawn. There is a need for more homogenous RCTs with large sample size to define the role of CHX in non-surgical management of peri-implant mucositis and peri-implantitis.

** Key words:**Peri-implant disease, disinfection, dental implants, oral hygiene, chlorhexidine.

## Introduction

Peri-implant mucositis and peri-implantitis are common pathological conditions affecting implants in function. The prevalence of ﻿peri-implant mucositis and peri-implantitis is estimated to be 19%-65% and 1%-47% respectively (1). While, peri-implant mucositis is a reversible inflammatory lesion that is confined to the surrounding implant mucosa, it may progress to peri-implantitis if left untreated (2). Peri-implantitis is usually diagnosed on the basis of progressive marginal bone loss, probing depths of 6mm and presence of bleeding on probing (BOP) (3).

The main causative factor for these conditions is believed to be pathogenous bacteria. However, the clinical course may vary depending upon a number of factors like previous history of periodontitis, smoking, systemic diseases, prosthetic errors etc (4). Due to structural similarity of peri-implant soft tissues and gingiva, a homogenous response to biofilm formation in the form of inflammatory cell infiltration is seen in both tissues. Studies have also established an identical cause-and-effect relationship after 3-weeks of plaque accumulation around teeth (gingivitis) and implants (peri-implant mucositis) (5). Additionally, the anaerobic gram negative bacterial flora of peri-implantitis has been found to be similar to that of periodontitis (6). In view of the homogeneity, treatment protocols used to treat gingivitis and periodontitis have been used for management of peri-implant mucositis and peri-implantitis respectively. While the primary line of treatment is disruption of biofilm and reduction of bacterial loads, additional surgical procedures and adjunctive therapies like use of chlorhexidine (CHX), triclosan based dentifrice, ﻿abrasive air blasting with sodium carbonate, photodynamic therapy and use of systemic antibiotics may be utilized depending upon the clinical condition (7,8).

﻿CHX is a commonly used topical agent for control and prevention of biofilm formation owing to its high substantivity, bactericidal activity and broad spectrum of action (9). However, owing to the implants’ macrostructure and surface characteristics, the biofilm content on an implant surface can be quite different from that of natural tooth surface (10,11). The implant surface has been shown to favor the presence of pathological bacteria even in the absence of peri-implant disease (10). In view of such differences, it is important to know if CHX has a role in managing peri-implant diseases. To date, a number of studies have evaluated the role of CHX in non-surgical treatment of peri-implant mucositis and peri-implantitis but with conflicting results (2,12,13). In the absence of clear clinical guidelines, the aim of this study was to systematically analyze literature and carry out a meta-analysis evaluating the role of CHX in improving outcomes with non-surgical management of peri-implant mucositis and peri-implantitis.

## Material and Methods

- Inclusion criteria and Search strategy

This systematic review and meta-analysis is based on the guidelines of the PRISMA statement (Preferred Reporting Items for Systematic Reviews and Meta-analyses) (14) and Cochrane Handbook for Systematic Reviews of Intervention (15). The research question to be answered was: Does local application of CHX improve outcomes in patients undergoing non-surgical treatment of peri-implant mucositis or peri-implantitis? The review protocol was prepared prior to initiation of the study.

An open-ended electronic search of articles published in the PubMed, Scopus, Embase, and CENTRAL (Cochrane Central Register of Controlled Trials) databases up to 1st August 2019 was carried out. Search words used in various combinations were: “chlorhexidine”, “peri-implantitis”, “peri-implant mucositis”, “dental implant”, “anti-microbial”, “anti-infective”, and “non-surgical”. The search strategy with results of PubMed database are presented in supplemental content 1. A hand search of references of included studies and relevant review articles was also carried out for identification of any additional studies.

PICOS (Population, Intervention, Comparison, Outcome, and Study design) outline was followed for identification of relevant articles. We included randomised controlled trials (RCTs) or controlled clinical trials (CCTs) conducted on adult patients (>18years) with peri-implant mucositis or peri-implantitis (Population); evaluating any form of local application of CHX (Intervention); comparing it with controls (Comparison) and assessing probing depth, BOP and/or clinical attachment levels (CAL) (Outcomes). We excluded in-vitro studies, studies on zirconia implants, animal studies, retrospective studies, single arm trials, case-series and non-English language studies. Studies comparing CHX with other active interventions (for e.g. laser therapy, air abrasive therapy), not studying any of the inflammatory outcomes (probing depths, BOP, CAL) and those evaluating the role of CHX with surgical treatment of peri-implant mucositis and peri-implantitis were also excluded.

- Data extraction and Outcomes

Literature search was performed by two independent reviewers. Title and abstracts of the retrieved studies were scrutinized, followed by full-texts evaluation of selected articles, for inclusion in the review. Disagreements were resolved by discussion to reach a definitive decision. Data was extracted by two independent reviewers using an abstraction form. The following details were collected: Authors, publication year, study type, sample size, selection criteria, treatment protocol, follow-up period and outcomes.

- Risk of bias

Cochrane Collaboration risk assessment tool for RCTs was used for quality assessment of the included trials (16). Studies were rated as low risk, high risk, or unclear risk of bias for: random sequence generation, allocation concealment, blinding of participants and personnel, blinding of outcome assessment, incomplete outcome data, selective reporting, and other biases.

- Statistical analysis

Review Manager (RevMan, version 5.3; Nordic Cochrane Centre [Cochrane Collaboration], Copenhagen, Denmark; 2014) was used for the meta-analysis. Meta-analysis was conducted only if at least 3 studies reported outcomes on the same scale. Anticipating heterogeneity amongst studies, a random-effects model was used to calculate the pooled effect size. Standardized Mean Difference (SMD) and Mean Difference (MD) with 95% confidence interval (CI) were used for pooling of continuous variables. Heterogeneity was calculated using I2 statistic. I2 values of 25-50% represented low, values of 50-75% medium and >75% represented substantial heterogeneity.

Meta-analysis of studies on peri-implant mucositis and peri-implantitis were carried out separately. Change of baseline scores were used for meta-analysis of studies on peri-implantitis. In studies where change scores were missing, the following equation was used for calculating the change in mean and standard deviation (SD) scores: Mean (Change)= Mean (After) - Mean (Before) and SD (Change)= square root {[SD2(after)- SD2(before)]/2}. A sensitivity analysis was carried out to assess influence of each study on the pooled effect size. Every study was eliminated sequentially to analyze any change in the results of the meta-analysis. Outcomes not pooled for a meta-analysis were presented in descriptive form.

## Results

**Figure 1:**
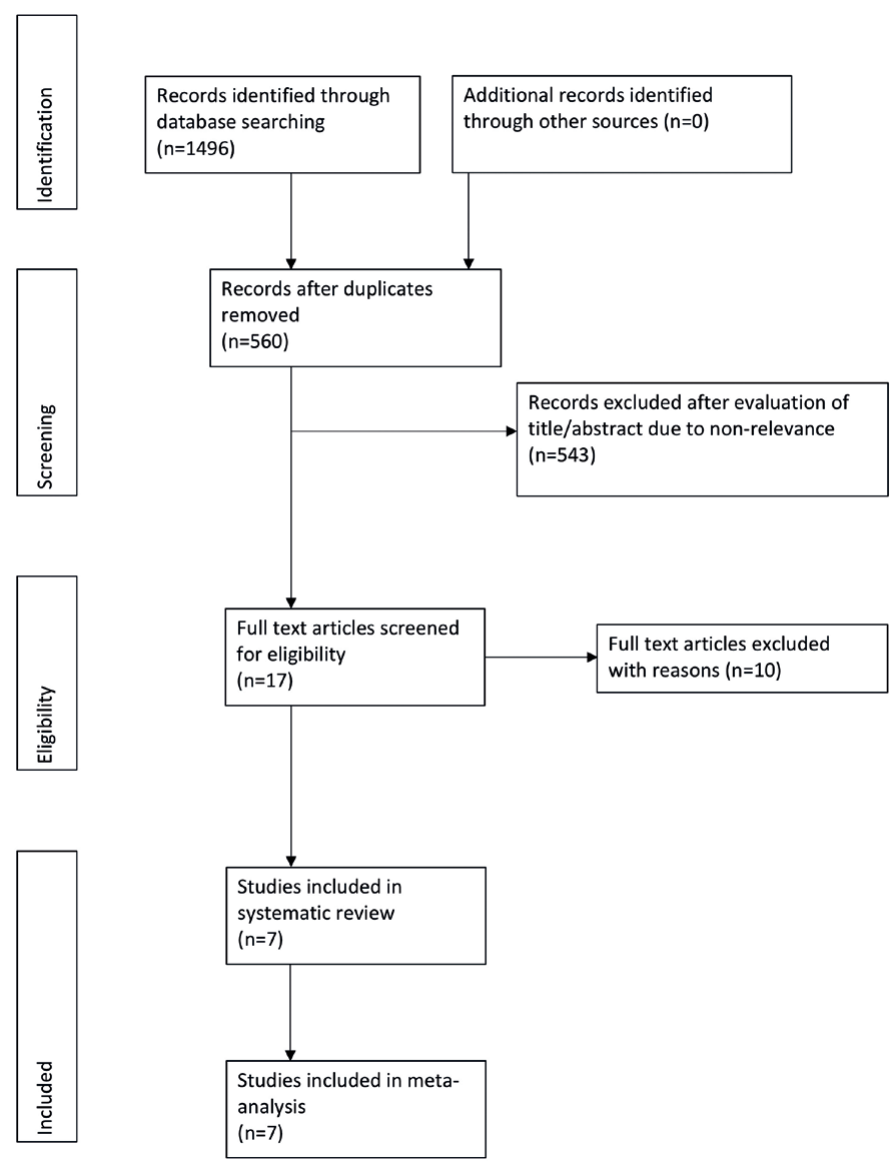
Study flow-chart.

Search results are presented in Fig. 1. Of the 17 studies selected for full-text evaluation, ten studies were excluded (17-26). Reasons for exclusion are presented in Table 1. A total of seven studies were included in the systematic review and meta-analysis (2,12,13,27-30).

**Table 1:**
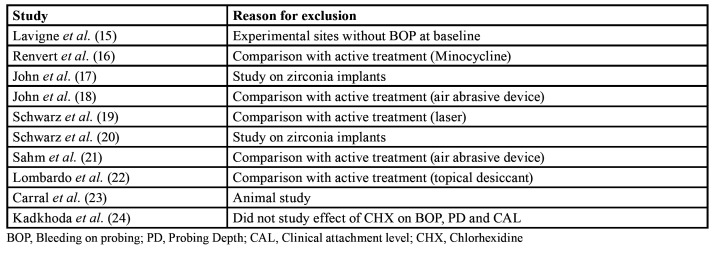
Reasons for exclusion of studies.

Baseline characteristics of included studies is presented in Table 2. Six were RCTs (2,13,27-30) while one was a CCT (12). Four studies (2,27-29) evaluated the role of CHX in peri-implant mucositis while three (12,13,30) assessed the drug in patients with peri-implantitis. All studies involved titanium implants. Patients were treated with various forms of CHX which included chips, gels, mouth rinses and irrigation devices. Oral prophylaxis with mechanical cleansing of implant surface prior to drug use was carried out in both groups and in all seven studies (2,12,13,27-30). However, CHX treatment protocol differed widely. The follow-up of included studies ranged from 1 month to 36 months.

**Table 2:**
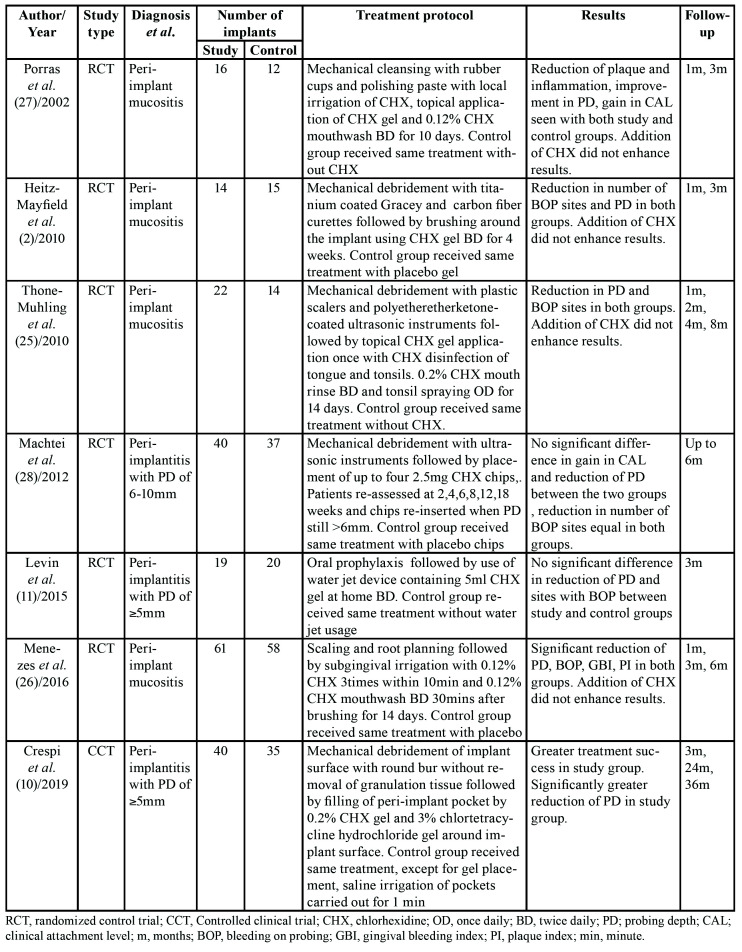
Characteristics of included studies.

Outcomes

- Probing Depth

**Figure 2:**
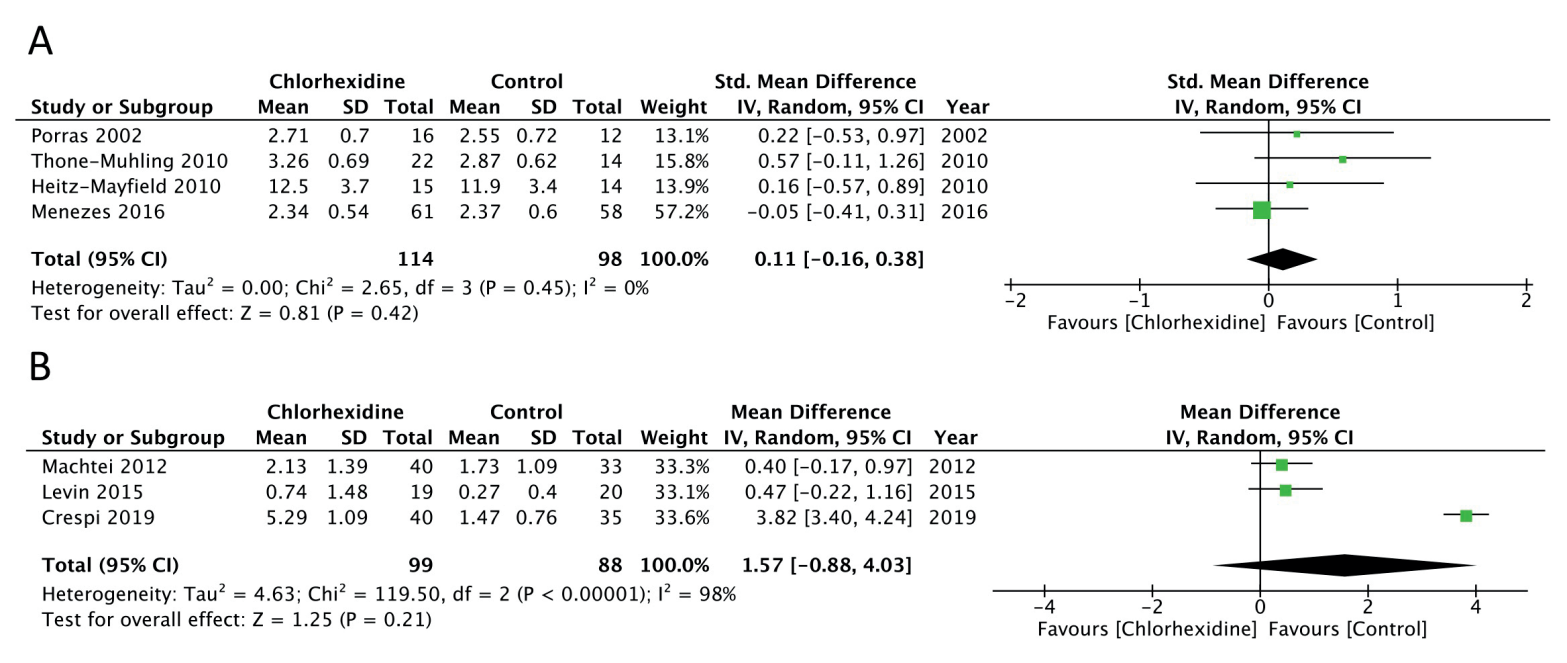
Forrest plot of probing depth A) for peri-implant mucositis B) for peri-implantitis.

Pocket probing depth around the implant was evaluated by all seven studies (2,12,13,27-30). Out of the four studies on peri-implant mucositis (2,27-29), three studies (27-29) reported mean probing depths around the implant while one (2) reported sum of the four probing sites around the implant. Meta-analysis indicated no difference in probing depths between the study and control groups at 2-3 months of follow-up (SMD= 0.11; 95% CI:-0.16 to 0.38; *p*=0.42, I2= 0%) (Fig. 2). Meta-analysis for probing depths in patients with peri-implantitis was carried out using change in baseline scores. Our pooled analysis failed to demonstrate any significant beneficial effect of CHX in improving probing depths in peri-implantitis at 3-6 months of follow-up (MD= 1.57; 95% CI:-0.88 to 4.0; *p*=0.21, I2= 98%) (Fig. 2). There was no change in the significance and direction of effect size on sensitivity analysis.

- Bleeding on Probing

Effects of CHX on BOP was studied by all seven trials (2,12,13,27-30). However, due to heterogeneity in reporting of data, meta-analysis could not be carried out. Results of individual studies are presented in descriptive form.

Porras *et al*. (29) in their cohort of peri-implant mucositis patients found as significant reduction of BOP sites in both study and control groups. There was no significant inter-group difference. However, data of individual groups was not presented in the article. Heitz-Mayfield *et al*. (2) in their study of 29 implants with peri-implant mucositis, found significant reduction of mean number of BOP-positive sites with CHX (from a baseline of 2.51 to 1.10.9 at 3 months) and placebo (from a baseline of 2.31 to 0.70.9 at 3 months). There was no statistical significant difference between CHX and placebo at 1 months and 3 months (*p*>0.10).

Thone-Muhling *et al*. (27) reported significant reduction of BOP positive sites with CHX (change of -0.090.09) as compared to control group (change of -0.130.17) at 2 months (*p*<0.05). Similar results were noted at 4 months but not after 8 months of follow-up. Menezes *et al*. (28) reported percentage of BOP positive sites (%BOP) around implants with peri-implant mucositis. They found statistical significant reduction of %BOP sites with CHX (75.8233.98 to 45.7634.85) and placebo (67.5434.38 to 41.0841) at 6 months of follow-up, however, there was no significant inter-group difference.

Machtei *et al*. (30) in their RCT on peri-implantitis, reported a significant reduction in %BOP with CHX (reduction of 57.57.92) and placebo (reduction of 45.58.8) with no significant inter-group difference at 6 months. On the other hand, Levin *et al*. (13) in their trial of peri-implantitis patients, reported significant beneficial effect of CHX water jet in reduction of BOP positive sites as compared to placebo at 3 months (*p*=0.011). Complete data was not available in the published article. Crespi *et al*. (12) in their RCT reported that addition of CHX to non-surgical therapy results in significantly greater reduction of %BOP sites (94.810.4 to 16.818.2) as compared to placebo (92.511.8 to 78.51.2%) at 3 months (*p*<0.001). Similar difference was seen at 36-months of follow up.

- Clinical attachment levels

Two studies reported the effect of CHX on CAL in peri-implant mucositis. In the trial of Porras *et al*. (29), statistical significant change in CAL was seen in both study and control groups. The authors concluded that addition of CHX did not significantly improve outcomes. Thone-Muhling *et al*. (27) reported significant improvement in CAL in control group but not in the CHX group after 8 months of follow-up.

Two trials reported CAL outcomes in peri-implantitis patients. Machtei *et al*. (30) reported significant increase in CAL with both CHX (2.210.23mm) and placebo (1.560.25mm) in patients with peri-implantitis at 6 months with no statistical significant difference between the two groups (*p*=0.05). Crespi *et al*. (12) reported a significant improvement of CAL from a baseline of 8.181.29mm to 3.670.81mm with CHX and a similar significant improvement from a baseline of 7.551.18mm to 6.691.43mm with placebo at 3months. However, outcomes with CHX were significantly better than placebo at 3months, 24 months and 36 months (*p*<0.001).

- Risk of bias assessment

**Figure 3:**
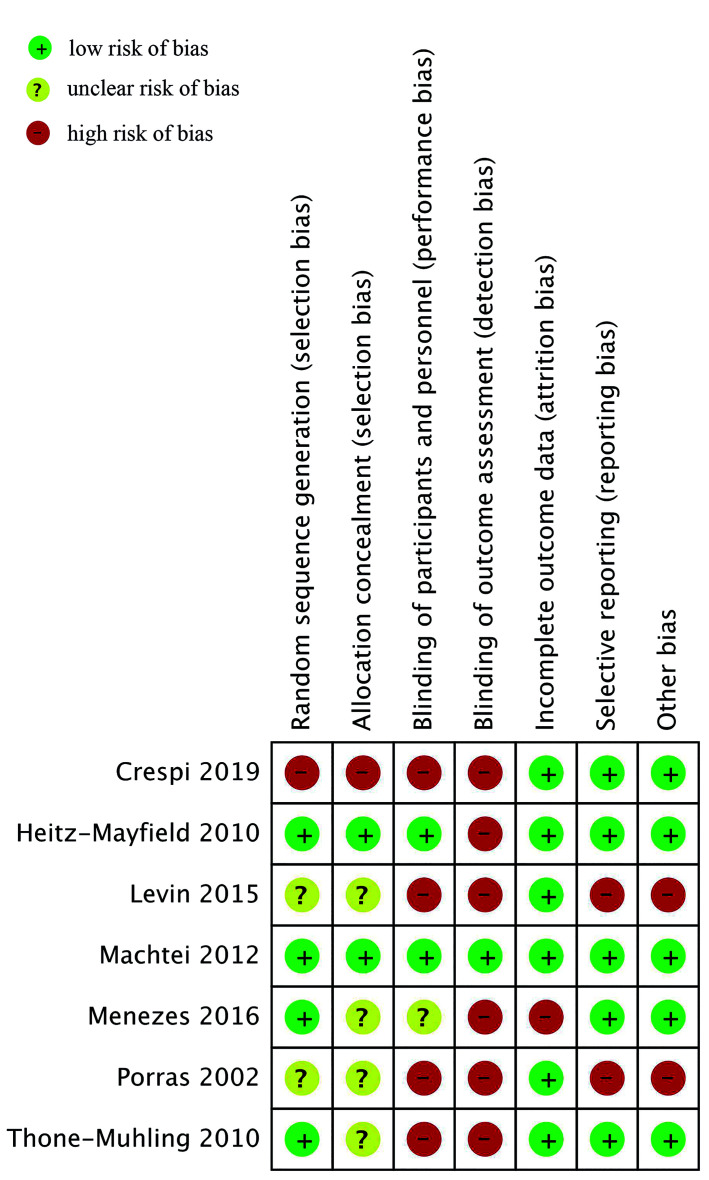
Risk of bias summary.

Authors judgement of risk of bias in included studies is presented in Fig. 3. Adequate method of randomization and allocation concealment was reported in four (2,27,28,30) and two trials (2,30) respectively. Blinding of personnel and participants was adequately reported in two studies (2,30) while blinding of outcome assessment was reported by only one trial (30).

## Discussion

The primary aim of this study was to evaluate whether supplementation of CHX with non-surgical therapy resulted in improved outcomes in the management of peri-implant mucositis and peri-implantitis. Paucity of RCTs and CCTs resulted in inclusion of only four studies for peri-implant mucositis and three studies for peri-implantitis in our systematic review.

Peri-implant mucositis in experimental conditions has been found to be reversible once oral hygiene measures are reinforced and biofilm is mechanically disrupted (31). Since peri-implant mucositis acts as a precursor to peri-implantitis, early management of this reversible process may reduce the incidence of peri-implantitis and implant failures (32). In search for an optimal management protocol for peri-implant mucositis, a number of adjunctive measures to mechanical therapy have been studied (7,8). In 2008, Renvert *et al*. (33) in a literature review concluded that while non-surgical mechanical therapy is effective in the management of peri-implant mucositis, addition of anti-microbial mouth rinses enhanced the outcomes of mechanical therapy for mucositis lesions. However, in 2015, Schwarz *et al*. (34) in a systematic review and meta-analysis concluded that adjunctive antiseptic, antibiotic (local and systemic) or mechanical therapy did not improve outcomes with mechanical therapy of peri-implant mucositis. While these previous reviews have been more generalized in their definition of “adjunctive therapies”, our study evaluated a specific adjunctive therapy: CHX. Our meta-analysis revealed that addition of CHX to mechanical therapy resulted in no difference in probing depths at short term follow up. Also, none of the four studies reported statistical significant difference in the number of BOP positive sites between the two groups. Two studies reported no difference in CAL with or without CHX. These results should be interpreted with caution as there was wide variation in the method of CHX delivery and total treatment time in the included studies.

Studies have shown that periodontopathogens may not be restricted to periodontal pockets alone, but they may also establish on tongue, tonsils and other oral mucosal sites (35). Therefore, Thone-Muhling *et al*. (27) studied the “one stage full-mouth disinfection” protocol of ﻿Quirynen *et al* in peri-implant mucositis wherein disinfection of tongue and tonsils were also carried out with CHX, unlike the other three included studies. Secondly, Felo *et al* (36) have suggested that CHX administered via powered sub-gingival irrigation may result in better outcomes than mouth rinses alone. CHX was used in gel from by Heitz-Mayfield *et al* (2), as a mouth rinse by Menezes *et al* (28), Thone-Muhling *et al* (27) and both gel and mouth-rinse by Porras *et al* (29). None of the included trials in our review studied the effect of subgingival CHX irrigation for peri-implant mucositis. Due to paucity of studies and limited sample size on included trials, the exact role of the mode of CHX delivery and extent of disinfection in influencing outcomes in peri-implant mucositis cannot be elicited.

While non-surgical management is considered to be effective for peri-implant mucositis, the optimal treatment protocol for the management of peri-implantitis is still debaTable. Renvert *et al*. (33) suggest that non-surgical therapy alone is not effective in the management of peri-implantitis. Fagglon *et al* (37) in their meta-analysis of eleven studies report that debridement in conjunction with antibiotics resulted in greater probing depth reduction than debridement alone. The role of CHX as an adjunctive therapy to non-surgical management of peri-implantitis is also unclear. Of the three studies included in our review, Crespi *et al* (12) reported significantly better success rates in the study group (100%) as compared to the control group (31.4%). This is in contrast with the studies of Machtei *et al* (30) and Levin *et al* (13) which reported no significant difference in results between CHX and control. The variation in outcomes may be explained by the different treatment methodology employed by Crespi *et al* (12). Addition of 3% chlortetracycline hydrochloride gel along with CHX around the implant surface may have reduced the bacterial load and detoxified the implant surface in their trial. The authors also hypothesized that leaving the granulation tissue in the soft tissue pocket may have resulted in proliferation of cells with embryonic stem cell properties thereby leading to better healing of tissues (38). With large variations in methodology amongst the three studies, definite conclusions on the role of CHX in non-surgical management of peri-implantitis cannot be drawn.

The lack of effectiveness of CHX as an adjunctive therapy for peri-implant disease as seen in majority of included studies may be explained by the difference in the drug’s substantivity between tooth and implant surfaces. While CHX demonstrates superior bonding to tooth surface, its adhesion on titanium depends upon the surface roughness and CHX concentration (39). Ryu *et al*. (40) have demonstrated that CHX adsorbed on the non-treated implant surface is rapidly released in 3 days while preparation of implant surface with sand blasting and acid etching may result in better CHX uptake. Due to a variety of implants and different drug concentrations used in the seven studies, the actual CHX adsorption and the duration of the following anti-microbial effect may have skewed results. New evidence also suggests that lack of better response after implant decontamination with chemotherapeutic agents may be due to alteration of implant surface by the drugs. Kotsakis *et al*. (41) have shown that CHX may alter the biocompatibility of implant surface and therefore should not be recommended for detoxification of implant surface. In light of this new evidence, further studies in-vitro studies need to be carried out to evaluate the effects of CHX on titanium surfaces.

The drawbacks of our review need to be mentioned. Foremost, only seven studies were available for inclusion with many trials of small sample size. Secondly, there were only two studies (2,30) with minimal risk of bias therefore the overall quality of evidence was not high. Thirdly, there was wide variation in the methodology of the included studies making comparisons difficult. Lastly, there were differences in the treatment protocol with different concentrations and forms of CHX tested for different durations. These variations may have influenced overall results.

In spite of the limitations, to the best of our knowledge, our study is the first systematic review and meta-analysis evaluating the role of CHX for peri-implant mucositis and peri-implantitis. The results of our study indicate that adjunctive therapy with CHX may not improve outcomes with non-surgical management of peri-implant mucositis. Conclusions with regards to its role in non-surgical management of peri-implantitis cannot be drawn. The present quality of evidence is weak due to limited studies and methodological heterogeneity. There is a need for high quality RCTs with homogenous methodology to further study the role of CHX as an adjunctive therapy for peri-implant mucositis and peri-implantitis.
